# The Use of Titanium Mesh in Guided Bone Regeneration: A Systematic Review

**DOI:** 10.1155/2019/9065423

**Published:** 2019-02-07

**Authors:** F. Briguglio, D. Falcomatà, S. Marconcini, L. Fiorillo, R. Briguglio, D. Farronato

**Affiliations:** ^1^Department of Biomedical, Dental and Morphological and Functional Images, University of Messina, Messina, Italy; ^2^Department of Surgical, Medical, Molecular and Critical Area Pathology, University of Pisa, Pisa, Italy; ^3^Associate Professor of Periodontology, Department of Biomedical, Dental and Morphological and Functional Images, University of Messina, Messina, Italy; ^4^Assistant Professor, Department of Medicine and Surgery, University of Insubria, Varese, Italy

## Abstract

Several techniques have been proposed for bone regeneration in patients with atrophic ridges. Nowadays, GBR represents the gold standard, and it allows obtaining sufficient bone volumes for a correct implant-prosthetic rehabilitation. Our goal is to perform a systematic review of the literature on the use of titanium meshes in GBR in order to evaluate the reliability of the procedure, the regeneration obtained, and the failures. Furthermore, we will evaluate the success and survival rate of the inserted implants. The selected articles concern vertical and/or horizontal regeneration of the alveolar ridge using titanium grids, in association or not with biomaterials, before and simultaneously with implant placement. Six articles were selected for the present review, including a total of 139 patients, 156 sites, and 303 implants. Titanium grids in combination with autogenous bone were used in 2 cases, 5 in combination with a mixture of autogenous bone and bone substitutes. The overall survival and success rates of implants were 98.3% and 85.25%, respectively. In conclusion, our review shows how the use of titanium mesh represented a predictable method for the rehabilitation of complex atrophic sites.

## 1. Introduction

The first condition to achieve success in implant therapy is insert fixtures into appropriate bone volumes; in fact, the presence of insufficient bone volumes negatively affects long-term prognosis and implant survival [1]. Nowadays, guided bone regeneration represents the gold standard in bone regeneration for implant placement and is the most documented technique in literature. The biological bases on which this technique is based derive from GTR of periodontal tissues, described for the first time by Nyman in 1980. A mechanical protection of the clot is performed by using a barrier membrane to allow the migration and proliferation of osteoprogenitor cells and to prevent soft tissue colonization of the defect [2, 3].

Membranes must have some characteristics such as biocompatibility, tissue integration, cell selectivity, and in some cases, space making ability as reported widely in the literature [4–10]. The barrier membranes are divided into two categories: absorbable and nonresorbable. The nonresorbable membranes are PTFE (expanded or high density) and titanium mesh. Titanium meshes are used for their space-making effect and are associated with the use of grafting materials [11, 12]. Furthermore, the exposure rate of titanium meshes is lower than that of PTFE membranes [13], and if the exposure occurs, it is not necessary to remove immediately the mesh because its pore structure allows a proper vascular supply to the underlying tissues without interfering with the blood flow, and moreover, the risk of superinfection is poor [14].

The purpose of this study is to perform a systematic review of the literature about the use of titanium meshes during bone regeneration techniques in order to evaluate the success rate of the procedure, survival and success rate of implant, and the predictability of this surgical technique.

## 2. Materials and Methods

A systematic review of the literature on PubMed database was performed, limiting the research to the articles published between 1998 and 2018. The research was also limited to dental journals and only to articles written in English. In the selected articles, a preimplant bone regeneration was performed through the use of particulate bone (autologous and/or heterologous) and titanium membranes. The following keywords were used: (1) titanium mesh; (2) titanium membrane; (3) bone regeneration titanium mesh; (4) GBR, mesh; (5) mesh membrane; (6) GBR, titanium membrane; (7) guided bone regeneration, mesh; (8) guided bone regeneration, titanium membrane.

### 2.1. Inclusion and Exclusion Criteria

- Only studies conducted on humans have been selected, excluding studies in vitro or on animal.
- Randomized and nonrandomized clinical studies, cohort studies, and case series have been included while case reports have been excluded. In addition, all studies with a number of sites treated less than 12 were excluded.
- Studies with strong smokers (>10 sig/day) or patients with previous major systemic diseases (such as tumor or congenital malformation) were excluded. Moreover, studies with concomitant interventions (for example, with simultaneous sinus floor elevation) were excluded.
- We selected only articles in which the regeneration was performed through particulate bone (autologous or mix of autologous and heterologous). The regeneration with bone block was excluded.
- Only articles using titanium meshes were included, eliminating all other forms of membranes (resorbable or nonresorbable).
- The minimum follow-up time is 6 months.
- Defect treated had nonspace making characteristics.

### 2.2. Study Selection

The research was conducted by two authors separately (DF and FB). The results have been compared at the end of the research. A possible disagreement regarding the inclusion of the studies was discussed among the authors.

**Figure 1:**
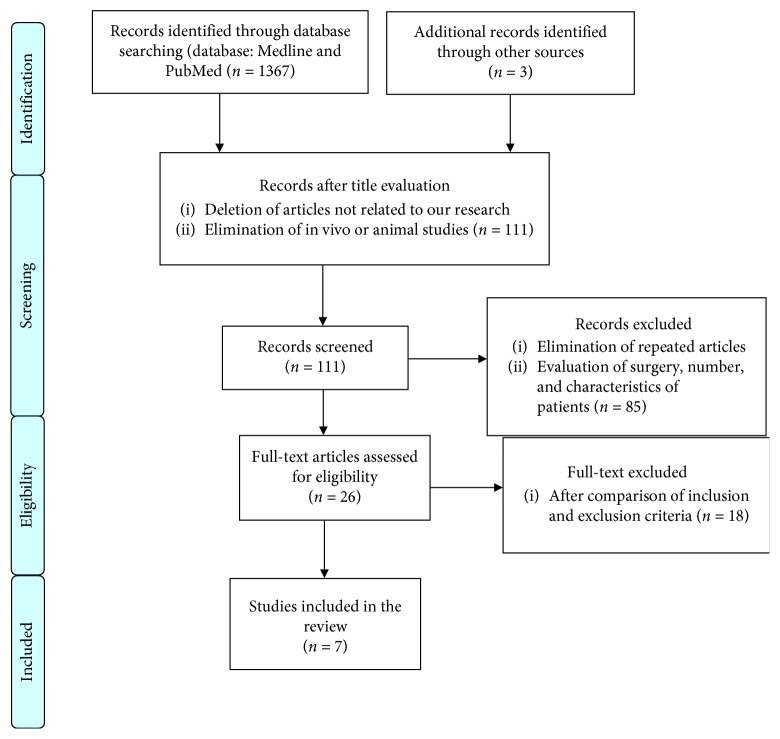
Flowchart (list of publications remained after full-text analysis and subsequent review).

The first phase of the research consisted of the selection of titles, which allowed us to make a first screening of the manuscript eliminating those not concerning our research, in vitro or animal study. After the first phase, we have reduced the number of articles from 1367 to 111. The second phase consisted in eliminating the repeated articles and reading the abstract of each article in order to evaluate some parameters of inclusion such as number and characteristics of the patients or the type of surgery performed. This second selection reduced the number of articles to 26. Finally, the full text of all studies was obtained and according to the expected inclusion/exclusion criteria. Seven articles were selected and included in the present review (Figure 1).

The implant success rate was evaluated according to Albrektsson et al.'s criteria [15]:

- Absence of persistent subjective complaints such as pain, foreign body sensation, and dysesthesia
- Absence of mobility
- Absence of peri-implant radiolucency and infection with pus suppuration
- Marginal bone resorption (MRB) not exceeding 1.5 mm after the first year of loading

## 3. Result

The selected articles are as follows: two retrospective studies, a case series, two clinical trials, two prospective studies, a retrospective longitudinal study, and a retrospective clinical study.

The total number of patients included in the selected studies was 154, with an average of 19.2 patients. The study with the highest number of patients recruited is Miyamoto et al. [16] while those with fewer patients are Lizio et al. [17] and Corinaldesi et al. [18], with a total of 12 patients. The female sex is slightly prevalent with 82 women and 74 men.

Since the same patient has been treated in several sites, the number of sites exceeds that of patients for a total of 175 sites treated and an average of 21.8. Both maxilla and mandible were treated in all studies, in particular 114 regenerated sites belong to the maxilla and 72 to the mandible.

In all studies, the graft used is composed of autologous particulate bone, associated with a heterologous bone. In particular, only in two articles [16, 19] autologous bone was used exclusively, in two cases autologous bone was associated with anorganic bovine bone (ABB) [17, 20], one case with hydroxyapatite [21], and in the remaining cases with BPBM and DBBM. In all the studies, the regeneration was carried out through the use of titanium mesh.

The average healing period was 7.5 months, with a range of 3 to 9 months. As regards osseointegration, instead, the expected times are comparable in all studies with 3 months of waiting for the fixtures inserted in the mandible and 4 months in the maxilla (Table 1).

In total, 348 implants were positioned, excluding Lizio in which the number of implants inserted is not provided. Uehara and Miyamoto report the loss of 1 implant (Table 2).

The complication found most frequently is the exposure of the mesh, with a total of 81 exposed meshes.

Poli et al. [22] obtained only one exposure after 4 months, treated with chlorhexidine rinses; Lizio obtained the exposure of 7 sites within the first 4–6 weeks treated with curettage and disinfection by chlorhexidine (0.2%). Other 5 exposures were delayed (after 4–6 weeks) and treated exclusively with chlorhexidine; the mean time of exposure was 2.17 months and a mean area of mesh exposure of 0.73 cm^2^. In the study of Proussaefs, the mesh was exposed within 2 weeks in two patients and after 3 months for 4 patients. Uehara obtained 16 exposed meshes (70%) of which 6 early (3-4 months) that were removed.

Miyamoto found the exposure of 8 mesh, of which 4 removed due to infection. Furthermore, Miyamoto reports partial bone resorption with minor infection in 5 cases and temporary neurological disturbances in 4 patients. Corinaldesi et al. [19] obtained 4 mesh exposures. Three meshes were exposed early (3–5 months) in patients treated with simultaneous approach. The fourth, however, was exposed in a patient with a two-step approach due to a periodontal infection of an adjacent natural tooth (Table 3).

Another important aspect is to evaluate the gain of bone after the regeneration procedure. Corinaldesi et al. [19] divided patients according to the surgical technique used. In patients treated with the simultaneous surgical technique, he obtained a mean height regeneration of 5.9 ± 1.77 mm at baseline.

However, peri-implant bone regeneration was 5.45 ± 1.81 mm. In patients undergoing a delayed approach, the mean vertical height was 5.5 ± 1.22 mm. The mean vertical bone gain was 4.5 ± 1.16 without statistically difference in vertical augmentation between the two groups (*t*-test, *P*=0.952).

Miyamoto divided the patients into 3 groups according to the type of defect, making a digital measurement of the bone gain. The mean augmented horizontal width was 4.3 ± 2.0 mm, and the vertical height was 8.1 ± 4.8 mm. In particular for the combined defect (horizontal + vertical), the mean horizontal gain was 3.7 ± 2.0 mm and mean vertical gain was 5.4 ± 3.4 mm. For the horizontal defects, the mean horizontal gain was 3.9 ± 1.9 mm. For the socket defects, the mean horizontal gain was 5.7 ± 1.4 mm and mean vertical gain was 12.4 ± 3.1 mm.

Proussaefs did three types of measurements: laboratory, radiographic, and histomorphometric. Volumetric laboratory measurements indicated 0.86 cc alveolar augmentation 1 month after bone grafting, 0.73 cc (SD 0.60) 6 months after bone grafting, and 0.71 cc 6 months after implant placement. Linear laboratory measurements indicated vertical augmentation of 2.94 mm 1 month after bone grafting, 2.59 mm 6 months after bone grafting, and 2.65 mm 6 months after implant placement. The corresponding measurements for labial-buccal augmentation were 4.47 mm, 3.88 mm, and 3.82 mm. Radiographic evaluation indicated 2.56 mm vertical augmentation and 3.75 mm labial-buccal augmentation. Histomorphometric evaluation indicated 36.47% new bone formation, 49.18% connective tissue, and 14.35% residual Bio-Oss particles.

Corinaldesi et al. [18] compared the use of only autologous bone (Control group) to a combination of autologous bone and BPBM for alveolar ridge augmentation (Test group). For the sites augmented only with particulate autologous bone, the amount of newly formed bone was 62.38% ± 13.02%, whereas connective tissue constituted 37.62% ± 13.02% of the entire area. For the sites augmented with a mixture of autologous bone and BPBM (test group), the amount of new bone was 52.88% ± 11.47%, the soft tissue was 29.96% ± 12.58%, and the remaining 17.16% ± 2.72% was filled with BPBM particles.

Lizio et al. [17] have made an assessment of the regenerated bone by means of 3D measurements, evaluating the lacking bone volume (LBV) and the planned bone volume (PBV). The mean LBV was 0.45 cm^3^ that was 30% of the mean PBV (1.49 cm^3^). Furthermore, evaluated how LBV was positively correlated with the area of mesh exposure.

## 4. Discussion

In the literature, there are few published studies concerning the regeneration of an atrophic site through the use of titanium meshes. Our systematic review aims to evaluate the results obtained in the last twenty years of regeneration making an assessment on three key points.

1. The regeneration obtained
2. Complications in particular exposure of the mesh, its eventual removal or loss of bone
3. The possibility of inserting implant fixtures and its success rate

The use of a titanium mesh in bone regeneration is of great importance, and the membrane in fact acts as a physical barrier that prevents the migration of epithelial cells and fibroblasts into the defect. This allows the osteoprogenitor cells to reach the site and recreate new bone. There are very few studies in the literature that relate the pore size on fibrous tissue ingrowth into porous barrier membranes and the consequent regeneration obtained. In an experiment carried out on rats, Salvatore et al. [23] examined the soft tissue response to polyurethane sponges in six pore sizes highlighting how reducing the pore size accelerates the growth of collagen and vascular tissue. Chvapil et al. [24] suggested that pores in excess of 100 *µ*m are required for the rapid penetration of highly vascular connective tissue, and small pores tend to become filled with more avascular tissue. A similar result was obtained by Taylor and Smith [25] who tested 2 types of porous methylmethacrylate implants, and they found that small pore size was inadequate for penetration of capillaries. Gutta et al. [26] in a randomized controlled study in dogs, analyzed three different pore sized meshes, and compared with controls without the mesh. They showed how macroporous membranes facilitated greater bone regeneration compared with microporous and resorbable membranes. Furthermore, macroporous mesh also prevented significant soft tissue ingrowth compared with other types of meshes.

In another study, Ari et al. assess two important properties of biomaterial: the pore size and hydrophobicity. As we said, the size of the pores can induce the formation of new blood vessels and improves the adhesion of progenitor cells to the regeneration material. Similarly, the degree of hydrophobicity of the material conditions cell adhesion and the speed of regenerative processes. In a study published in 2017, researchers evaluated the influence of chitosan, hydroxyapatite, and gelatine scaffolds with these two important properties. In particular, three scaffolds have been created with different ratios of constituents. The results showed that as the amount of hydroxyapatite increased, the pore size was reduced. However, the increase in chitosan-gelatine reduced the hydrophobicity of the material [27].

A fundamental role in regeneration is played by the ability of the biomaterial to act as a scaffold, in order to provide a mechanical structure to support cells, their growth, and differentiation and the formation of new bone tissue. Collagen has been used as a scaffold due to its biocompatibility and its excellent mechanical properties; today, it is considered one of the best and most promising materials for the future. In a recent study, the ability of collagen to stimulate the formation of VEGF (vascular endothelial growth factor) in regenerative surgery was assessed. VEGF can stimulate angiogenesis which is a key event in the bone regeneration process. The researchers selected 6 rats and divided into two groups. The first group was treated with the application of a scaffold placebo, while the second group had a collagen scaffold previously taken and treated. Histological results show a higher expression of VEGF in the test group rather than in the control group, thus increasing the process of angiogenesis and bone regeneration [28].

Nowadays, in bone regeneration, the use of a membrane to protect the graft represents the gold standard. Numerous studies in the literature demonstrate its effectiveness. Buser et al. [29] report how the use of a membrane to protect the graft reduces bone resorption. The same result is obtained by Antoun et al. [30] which showed a reduction in bone resorption in the regenerated sites with membrane and graft compared to those where the graft was used without the protection of the membrane. Cordaro et al. [31] demonstrated a reduction in vertical regeneration of 40% at 5 months, if the graft was not protected by a membrane.

The use of a resorbable rather than nonresorbable membrane certainly has advantages, for example, a greater handling. At the same time, with nonresorbable membranes, the risk is the displacement during the wound closure, or membrane collapse during healing with the consequent reduction of the space necessary for bone regeneration. Furthermore, resorbable membrane may cause the blockage of the periosteal blood supply by ingrowth of the angiogenic cells with slow healing [32]. The introduction of nonresorbable membranes has drastically changed the surgical techniques, increasing the regenerative capacity and improving the results of surgery [33].

The use of a nonresorbable Ti-mesh allows us to provide a shape and to maintain space between the membrane and the defect. Moreover, the presence of the pores permits to maintain a blood support both to the mucosa and to the bone during the regeneration phase. The presence of pores in fact facilitates metabolic processes and tissue nutrition. This was demonstrated in a study by Celletti et al. [34] in which using a titanium pore-free membrane obtained the exposure of all the meshes in three weeks.

The main complication related to the use of titanium membrane is the dehiscence of soft tissues with the consequent exposure of the mesh. Nonetheless, titanium meshes are able to tolerate a certain degree of exposure. Louis et al. [35] still obtained the exposure of 23 meshes on 44 treated patients (52%); only one case had failure of the graft with a success of the bone grafting procedure that was 97.72%. In contrast, in a study conducted by Maiorana et al. [36], the exposure of the mesh led to an early resorption of the site between 15% and 25%, which however allowed placing the implant fixtures. The rate of exposure of the Ti-mesh varies from 5.3% [37] to 52% [35] depending on the studies, despite that the exposure does not affect the implant results [38, 39].

Miyamoto et al. and Louise et al. [16, 35] show how the volume of regenerated bone must be related to the morphology of the defect. Complex defects (horizontal and vertical) are related to greater bone loss associated with exposure and lower bone gain. Similar results were obtained by Her et al. [14] and Proussaefs and Lozata [20], and the exposed area was associated with bone loss and reduced bone formation.

The literature reports several techniques to reduce the rate of membrane exposure, such as the application of platelet-rich plasma to the mesh [39] or the creation of customized meshes using CAD/CAM technology in order to fit perfectly to the site and reduce usage times. As regards, the thickness of the membrane the most used is 0.2 mm, since on the one hand it provides a rigidity sufficient to maintain the space between membrane and the site to be regenerated, farther it protects the graft. At the same time, this thickness gives a flexibility that reduces the risk of soft tissue dehiscence.

The capacity for bone regeneration through the use of a titanium mesh does not have precise values. The maximum vertical regeneration obtained with simultaneous implant placement was 13.7 mm [20], but in general, the average for vertical regeneration ranging from 2.56 to 6 mm [19, 35]. The horizontal regeneration instead is on average 4 mm as reported by numerous studies [16, 19, 35, 38].

Most important factors that may limit regeneration are soft tissues, in fact especially in atrophic sites and in vertical increments, they can negatively affect the result. The proximity to muscle insertions and the lack of keratinized mucosa are factors that influence the mobilization of the flap and therefore increase the risk of dehiscence. The correct management of soft tissue might improve the effectiveness of the procedure.

A further consideration must be made on the measurement method of the bone regenerated in the various studies. Most relies on a linear measurement obtained either through the periodontal probe or radiographically on CT. A further consideration must be made on the measurement method of the bone regenerated in the various studies. Most relies on a linear measurement obtained either through the periodontal probe [19, 40] or radiographically on CT [16, 18, 38]. Surely evaluation by CT is more appropriate as it allows a better evaluation of bone gain/loss especially in complex defects. Only Proussaefs end Lozada calculated the regeneration obtained by the impression of the treated site before and after surgery. The mean added volume of bone was 0.86 cm^3^ at one month after surgery and 0.71 cm^3^ at six months after surgery.

Nevertheless, this method could not be optimal because it does not consider the thickness of the mucosa that varies considerably.

A characteristic found in the studies reported in this review is the presence under the mesh of a layer called “pseudoperiostium” [41]. This thin layer is evident when the mesh is removed, and it is made up of connective and granulation tissue. The clinical significance is still unknown. The literature reports how it is present beneath nonresorbable membranes [29, 33], while it is absent below collagen-absorbable membranes [42].

## 5. Conclusion

In the present systematic review, it is possible to assess how the regenerative procedures performed through the use of autologous and heterologous particulate grafts associated with a titanium mesh represented a predictable method for the rehabilitation of complex atrophic sites. The implant survival and implant success values obtained can be overlapped with those obtained by inserting implant fixtures in native bone. Nevertheless, the use of the titanium grids has disadvantages, for example, the necessity of a second surgical step increases the morbidity for the patient; furthermore, it has a risk of soft tissue dehiscence and membrane exposure. In this case, if this complication appears, the optimal management of membrane exposition permits to obtain a sufficient bone regeneration volume in a good cases percentage. So, a proper soft tissue management and careful preoperative examination nowadays make this technique the gold standard in regenerative surgery.

## Figures and Tables

**Table 1:** Evaluation of the horizontal/vertical bone regeneration.

	Number of patients	Number of sites	Graft	Type of augmentation	Vertical bone augmentation	Horizontal bone augmentation	Follow-up
Lizio et al. [17]	12	15	Autologous + ABB 70 : 30	V + O			
Corinaldesi et al. [18]	12	12	Autologous + BPBM 70 : 30	V			12 m
Corinaldesi et al. [19]	24	27	Autologous	V + O			3–8 y
Poli et al. [22]	13	13	Autologous + DBBM 1 : 1	V + O			88 m
Proussaefs and Lozada [20]	16	16	Autologous + ABB 1 : 1	V + O	V + O 8.1 O 5.4 S 12.4	V ∗ O 4.3 O 3.7 S 5.7	6 m
Miyamoto et al. [16]	41	50	Autologous	V + O	2.56	3.75	47.5 m
Uehara et al. [21]	21	23	Autologous + idrossiapatite 50 : 50	V + O			40 m
Tot.	139	156					

DBBM: demineralized bovine bone mineral; ABB: inorganic bovine bone; BPBM: bovine porous bone mineral.

**Table 2:** Evaluation of implant procedure.

	Implant	Implant surface	Implant lost	Bone loss	Success rate (%)	Survival rate (%)
Lizio et al. [17]						
Corinaldesi et al. [18]	35	Xive plus/spling twist mtx				100
Corinaldesi et al. [19]	56	Spline twist mtx			96.4	100
Poli et al. [22]	20		0	1.7 mm mesial 1.9 mm distal	100	100
Proussaefs and Lozada [20]	41	Idrossiapatite root form implant (nobel)				
Miyamoto et al. [16]	87		1		88	92.8
Uehara et al. [21]	64		1		56.6	98.8
Tot.	303		2			
Mean					85.25	98.32

**Table 3:** Evaluation of titanium mesh exposure.

	Mesh exposure	% mesh exposure	Mesh removed	Bone loss	Remotion time	Types of meshes used
Lizio et al. [17]	12	80	—	Yes		Ti mesh (ridge-form mesh; OsteoMed) 0.2 mm thick
Corinaldesi et al. [18]	0	0	—			Ace titanium micromesh, ACE surgical supply, Brighton Modus 1.5 mesh, Straumann
Corinaldesi et al. [19]	4	14.8	3		3–5 mesi	Ace titanium micromesh, ACE surgical supply company Modus 0.9 mesh, Medartis
Poli et al. [22]	1	7.69	—			0.2 mm thick Ti-Mesh (KIS Martin, Tuttlinger, Germany)
Proussaefs and Lozada [20]	6	35.29	—	Yes		Mesh (Osteo-Tram; OsteoMed)
Miyamoto et al. [16]	18	36	4	Yes		0.1 and 0.2 mm thickness; M-TAM, Stryker Leinger GmbH & Co., KG, Freiburg ASTM F-67 Jeil Medical Corp., Seoul, Korea
Uehara et al. [21]	16	70	6	Yes	3–7 mesi	0.3 mm thick microtitanium mesh (Striker-Leibinger), Freiburg, Germany 1.4 HOMS Engineering, Chino, Japan
Tot.	57		13			
Mean		34.8	22.8			
